# ECMO in the Management of Noncardiogenic Pulmonary Edema with Increased Inflammatory Reaction After Cardiac Surgery: A Case Report and Literature Review

**DOI:** 10.3390/diseases12120316

**Published:** 2024-12-04

**Authors:** Raluca Elisabeta Staicu, Ana Lascu, Petru Deutsch, Horea Bogdan Feier, Aniko Mornos, Gabriel Oprisan, Flavia Bijan, Elena Cecilia Rosca

**Affiliations:** 1 Doctoral School Medicine-Pharmacy, “Victor Babes” University of Medicine and Pharmacy Timisoara, Eftimie Murgu Square No. 2, 300041 Timisoara, Romania; raluca.staicu@umft.ro; 2 Institute for Cardiovascular Diseases of Timisoara, Clinic of Anesthesia and Intensive Care, “Victor Babes” University of Medicine and Pharmacy Timisoara, Gheorghe Adam Street, No. 13A, 300310 Timisoara, Romania; deutsch.petru@umft.ro (P.D.); mornosaniko@gmail.com (A.M.); oprisan_89@yahoo.com (G.O.); flavia.bijan@yahoo.com (F.B.); 3 Institute for Cardiovascular Diseases of Timisoara, “Victor Babes” University of Medicine and Pharmacy Timisoara, Gheorghe Adam Street, No. 13A, 300310 Timisoara, Romania; 4 Department III Functional Sciences—Pathophysiology, “Victor Babes” University of Medicine and Pharmacy of Timisoara, Eftimie Murgu Square No. 2, 300041 Timisoara, Romania; 5 Centre for Translational Research and Systems Medicine, “Victor Babes” University of Medicine and Pharmacy Timisoara, Eftimie Murgu Square No. 2, 300041 Timisoara, Romania; 6 Department of Surgery X, “Victor Babes” University of Medicine and Pharmacy Timisoara, Eftimie Murgu Square No. 2, 300041 Timisoara, Romania; horea.feier@umft.ro; 7 Advanced Research Center of the Institute for Cardiovascular Diseases, “Victor Babes” University of Medicine and Pharmacy Timisoara, Eftimie Murgu Square No. 2, 300041 Timisoara, Romania; 8 Department VI Cardiology—Cardiovascular Surgery, “Victor Babes” University of Medicine and Pharmacy Timisoara, Eftimie Murgu Square No. 2, 300041 Timisoara, Romania; 9 Department of Neurology, “Victor Babes” University of Medicine and Pharmacy Timisoara, 300041 Timisoara, Romania; rosca.elena@umft.ro; 10 Clinical Emergency County Hospital Timisoara, 300736 Timisoara, Romania

**Keywords:** noncardiogenic pulmonary edema, inflammation, deep vein thrombosis, ECMO

## Abstract

Noncardiogenic pulmonary edema after cardiac surgery is a rare but severe complication. The etiology remains poorly understood; however, the issue may arise from multiple sources. Possible causes include a significant inflammatory response or an autoimmune process. Pulmonary edema resulting from noncardiac etiologies can necessitate extracorporeal membrane oxygenation (ECMO) because most of the cases present a substantial volume of fluid expelled from the lungs and the medical team must manage the inability to achieve effective ventilation. A 64-year-old patient with known heart disease was admitted to our clinic with acute pulmonary edema. His medical history included Barlow’s disease, severe mitral regurgitation (IIP2), moderate–severe tricuspid regurgitation, and moderate pulmonary hypertension. The patient had a coronary angiography performed in a prior hospitalization before the surgical intervention which indicated the absence of coronary lesions. Preoperative screening (nasal, pharyngeal exudate, inguinal pouch culture, and urine culture) was negative, with no active dental infections. The patient was stabilized, and 14 days post-admission, mitral and tricuspid valve repair was performed via a thoracoscopic approach. After being admitted to intensive care post-surgery, the patient quickly developed pulmonary edema, producing a large volume (4.5 L) of yellow secretions through the intubation tube followed by hemodynamic instability necessitating high doses of medications to support circulation but no cardiorespiratory arrest. Due to his worsening condition, the patient was urgently taken back to the operating room, where veno-venous extracorporeal membrane oxygenation (VV-ECMO) was initiated to support oxygenation and stabilize the patient.

## 1. Introduction

The cases described in the specialized literature of noncardiogenic pulmonary edema after cardiac surgery have an unfavorable evolution without ECMO, accompanied by a very high mortality rate [1,2,3].

Due to its rarity and severity, this complication is difficult to treat and has a high mortality rate [4]. It is impossible to ventilate the lungs due to a massive accumulation of fluid expelled from the lungs through the intubation tube, leading within minutes to hemodynamic instability. This fluid does not have the appearance of the aerated, reddish sputum from acute cardiogenic pulmonary edema, but rather like a yellowish plasma-like liquid which is externalized in a very significant amount. Drawing from practical experience and studies, it is known that the occurrence of this type of pulmonary edema following cardiac surgery, in particular after thoracoscopic interventions for mitral valve repair, is difficult, if not nearly impossible, to treat without VV-ECMO [2]. Rapid recognition of this life-threatening refractory respiratory insufficiency led to the institution of VV-ECMO [5]. In cardiac surgery, accurate diagnosis within a short time may be challenging in intriguing circumstances like the incidence of sudden noncardiogenic pulmonary edema in patients with ventricular disfunction. In these cases, the medical team needs to react quickly to make the best decision and prevent the right treatment from being delayed.

Two forms are described in the specialized literature: noncardiogenic pulmonary edema (NCPE) with the involvement of both lungs, and unilateral noncardiogenic pulmonary edema (UPE). UPE is more frequent (5.6% to 25%) after mitral valve plasty through a thoracoscopic approach [6]. Transfusion, prolonged cardiopulmonary bypass (CPB) time, chronic lung diseases, an elevated preoperative inflammatory profile, sepsis, protamine administration, paraneoplastic syndrome, anesthesia, hypothermia, surgery, medications, inflammatory hyperreactivity, or even a hyperimmune response are some of the risk factors that have been identified in the literature, while the causes of this complication remain unknown [1,2,3,7]. This study and case report aim to demonstrate the severity of noncardiogenic pulmonary edema, the significance of starting therapy as soon as possible, and whether inflammation plays a significant role in this problem. We intend to increase our understanding of this issue by collecting data from multiple heart surgery clinics, to observe the ordering strategy and type of treatment in this pathology.

## 2. Materials and Methods

To identify other articles that presented noncardiogenic pulmonary edema and especially the one that occurred after mitral valve repair by a thoracoscopic approach, we performed a search of the international medical literature in the last 12 years in Google Scholar, PubMed, and Web of Science. We also performed a search in our review for patients who presented this type of pathology, and we selected 11 studies from the literature with the aim of highlighting the diagnosis, treatment, and evolution of this pathology. We shared the data collected in the introductive part and in the Discussion Section.

This study was conducted in accordance with the Declaration of Helsinki, and approved by the Institutional Review Board of Institute of Cardiovascular Diseases (protocol code 2098/16 March 2022) and the Ethics Committee of Victor Babes University of Medicine and Pharmacy Timisoara (protocol code 91/29 April 2022).

## 3. Results

A 64-year-old male was admitted to our clinic exhibiting acute pulmonary edema, dyspnea, tachypnea, and rales upon examination, accompanied by hypoxia, oliguria, and elevated blood pressure readings. After hemodynamic and respiratory stabilization, the microbiological screening was attained and 14 days later the patient underwent the surgical intervention of mitral and tricuspid valve plastic surgery through a thoracoscopic approach. The diagnostic workup included a reevaluation of the coronary angiography image results that the patient had 14 days before his emergency admission into our clinic, which showed the normal origin of the dominant right coronary artery in the right coronary sinus of the aorta and the normal origin of the left main coronary artery from the left coronary sinus of the aorta with no obstructive epicardial coronary artery disease, and a transthoracic ultrasound that revealed Barlow’s disease, severe mitral regurgitation (IIP2), moderate–severe tricuspid regurgitation, and moderate–severe pulmonary hypertension. The systolic function of the left ventricle (LV) was normal measured with Simpson’s biplane method (ejection fraction = 55%, calculated with the formula: Vtd-Vts/Vtdx100), with an end-diastolic volume (EDV) = 160 mL; left atrium (LA) diameter = 5 cm; mitral annulus (MA) length (A2) = 3.1 cm; MA length (P2) = 2.4 cm; and dilated mitral ring = 4.5 cm.

Please refer to Table 1 for the patient’s preoperative laboratory results: white blood cell count: 7.98/103/µL; high-sensitivity C-reactive protein: <5 mg/L; and procalcitonin (PCT): negative.

Blood gas analysis during nasal cannula oxygen therapy (3 L/min) revealed normal values. Chest X-ray was unremarkable.

**Figure 1:**
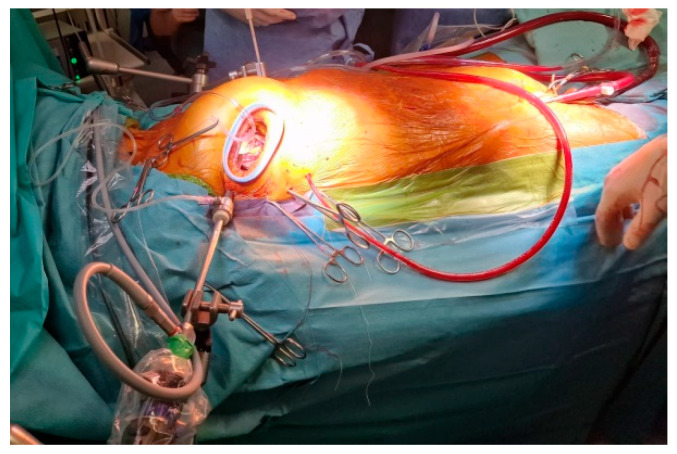
Minithoracotomy mitral valve repair.

**Figure 2:**
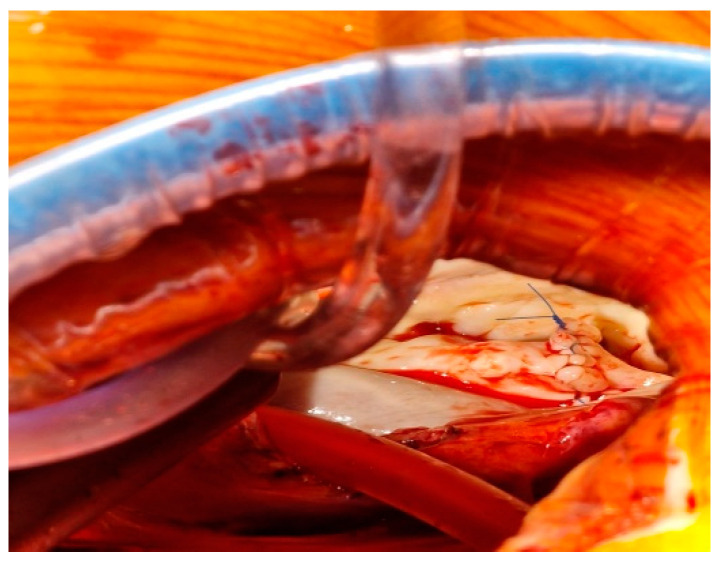
Intraoperatory aspect of mitral valve repair.

The surgery proceeded involving mitral and tricuspid valve repair through a thoracoscopic approach (Figure 1 and Figure 2).

**Figure 3:**
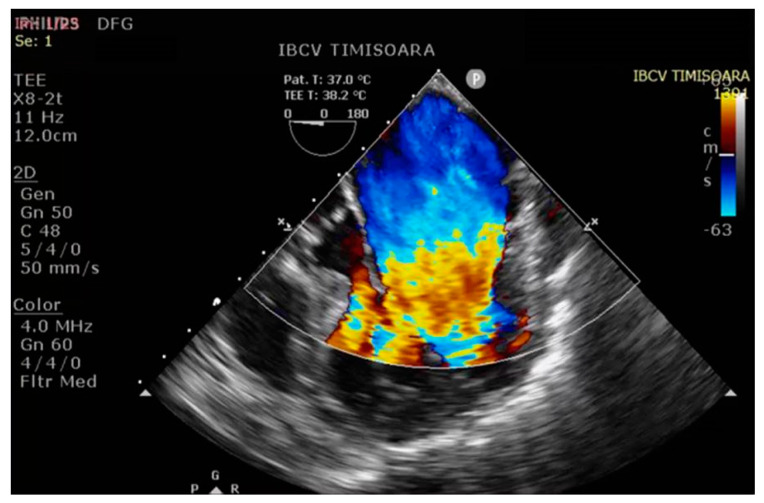
Color Doppler aspect of the repaired mitral valve.

**Figure 4:**
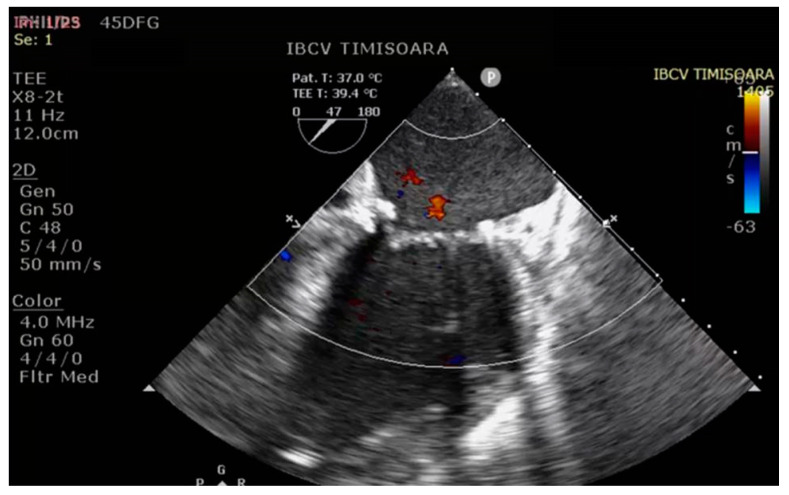
Post−operative aspect of the mitral valve (2D echocardiography).

The cardiopulmonary bypass (CPB) time was 175 min, with an aortic cross-clamp time of 102 min and ultrafiltration of 5500 mL. After the completion of CPB, the arterial blood pressure was 120/50 (73) mmHg, the central venous pressure was 6 cmH_2_O, the heart rate was 80 bpm, and the peripheral blood oxygen saturation was 92% (ventilation parameters synchronized by intermittent mandatory ventilation (SIMV), tidal volume (TV) = 400 mL, RR = 14, FiO_2_ = 0.7, positive end-expiratory pressure (PEEP) = 6 cmH_2_O). Postoperatively, transesophageal echocardiography (TEE) revealed normal cardiac contractility and repaired heart valves (Figure 3 and Figure 4).

Following admission to intensive care, the patient’s respiratory and hemodynamic condition began to decline with severe decompensated respiratory and metabolic acidosis (arterial blood gasses sample values: pH = 7.00, p CO_2_ = 65 mmHg, p O_2_ = 68 mmHg, c HCO_3_^−^ = 12.2 mmol/L, BE = −16 mmol/L, cSO_2_ = 81%, Lactate (Lac) = 8 mmol/L), necessitating volume administration, augmented doses of vasopressors, and inotropic support. Despite the initial treatment, the patient remained hemodynamic instable and required volume and vasopressors boluses to sustain a mean arterial pressure (MAP) range of 30–40 mmHg (but not cardiorespiratory arrest). Emergency repeated transthoracic echocardiography revealed normal cardiac contractility. Due to the patient’s circulatory condition and ventilation difficulties, transfer to the operating room was conducted rapidly. Mechanical ventilation, alternatively ARDS-like ventilation, was required due to the evacuation of a large volume of yellow liquid (4.5 L—plasma-like) on the intubation tube. Since the postoperative transthoracic ultrasound revealed no cardiac failure and the diagnosis was acute pulmonary edema (yellowish secretions in the intubation tube, increased airway pressures on the ventilator, and sudden hypoxia, with rales in both lung regions), ruling out a mechanical cause was the first choice of the medical team. After sternotomy, exploring the mediastinum, and excluding a mechanical cause, the decision to initiate VV-ECMO was made because, despite optimized ventilation with PEEP = 14 cm H_2_O (PEEPi = 9 cmH_2_O) [8] measured by an expiratory hold maneuver, FiO_2_ = 1, TV = 350 mL, RR = 18/min, compliance (mL/cmH_2_O) = 8, and resistance (cmH_2_O/L/s) = 80, adequate oxygenation could not be achieved.

Following the initiation of veno-venous extracorporeal membrane oxygenation (VV-ECMO), there was a modest improvement in the patient’s blood gas parameters and hemodynamic stability, reflected in an elevation of blood pressure and a decreased need for vasopressor and inotropic support. A percutaneous 25 Fr cannula was inserted in the right femoral vein via the previous groin incision and advanced to the level of the right atrial–inferior vena cava junction. Another 23 Fr cannula was inserted in the superior vena cava via direct cannulation and placed at the right atrial level. The superior vena cava cannula was placed at the upper edge of the incision, allowing closure of the sternum. The post-ECMO arterial blood gas analysis revealed the following values: pH 7.15, p CO_2_ 48 mmHg, p O_2_ 78.5 mmHg, bicarbonate (c HCO_3_^−^) 17.2 mmol/L, base excess (BE) −11.5 mmol/L, oxygen saturation (c SO_2_) 91.2%, and lactate (Lac) 5.6 mmol/L. The vasopressor agents administered included adrenaline at 0.05 μg/kg/min and norepinephrine at 0.1 μg/kg/min. Subsequently, the patient was switched to the intensive care unit for ongoing critical care management.

Regarding ventilation, the patient was switched to the airway pressure release ventilation (APRV) ventilation mode (established parameters: PEEP = 10 cmH_2_O, RR—10/min, P_plat_ < 25 cmH_2_O, P_peak_ < 25 cmH_2_O). (See Table 2).

An enhancement in blood gas parameters was observed, accompanied by an increased tidal volume (TV), improved pulmonary compliance, and a reduction in pulmonary resistance.

**Figure 5:**
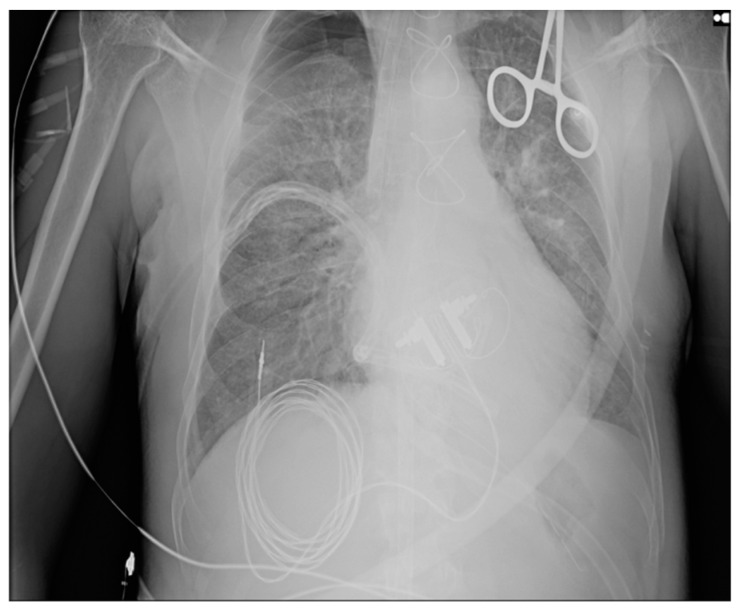
Chest X-ray suggestive of ARDS.

**Figure 6:**
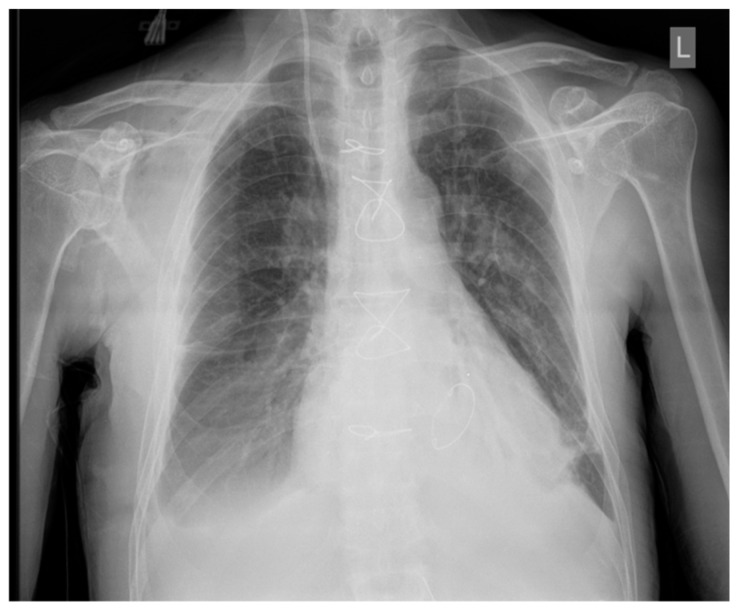
Chest X-ray at 3 days of VV-ECMO, suggestive of a favorable outcome of the alveolar infiltrates.

Daily chest X-rays were performed. On Day 1, there was a massive alveolar infiltrate in both lung fields, except the right lung apex, with a sketched air bronchogram [4], the aspect advocated for ARDS (Figure 5). The evolution of pulmonary infiltrates was favorable after 3 days (Figure 6).

The patient benefited from VV-ECMO for 4 days. The blood tests during this period are presented in Table 3. The weaning parameters of ECMO and ventilation are displayed in the Supplementary Material, Table S1. In addition, due to a febrile episode, several cultures were collected: bronchial aspirate, blood culture, urine culture—all of which yielded negative results; this febrile episode was attributed to Systemic Inflammatory Response Syndrome (SIRS) because the patient presented specific criteria: leukocytosis, fever, and escalation of vasopressors during ECMO. However, it is difficult to establish if the SIRS was caused by the underlying disease or the presence of the ECMO circuit, because it is well known that extracorporeal life support is associated with an increased inflammatory reaction.

The patient was stable during ECMO and, after five days, weaning was accomplished.

On the seventh day of hospitalization in the intensive care unit (ICU), the patient developed hypoactive delirium that resolved in 48 h. In addition, on the 10th day of hospitalization in ICU, he developed deep vein thrombosis, which resolved under treatment with continuous-dose heparin (D-Dimer 1980 ng/mL).

Although the preoperative inflammatory profile showed normal values of high-sensitivity C-reactive protein, the neutrophil/lymphocyte ratio, and also PCT, after surgery a marked increase in the monitored markers was observed (Table 4).

The very high values of interleukin-6 (IL 6), suggestive for an immune hyperactivity, may show that the preoperative status of the patient (patient who had pulmonary edema, an chronic severe mitral regurgitation) could influence the marked increase in IL-6 in the postoperative period and favor the occurrence of noncardiogenic pulmonary edema. In addition, many studies show that in chronic lung disease with pulmonary hypertension, there is an association with increased levels of IL-6 [5]. A preoperative determination of the IL-6 value would have been particularly valuable, it would also be useful for all patients with a high risk of developing this complication.

## 4. Discussion

Establishing the diagnosis of noncardiogenic pulmonary edema and differentiating it from pulmonary edema due to cardiac causes is especially important because it requires a different approach from a therapeutic point of view. The conducted studies show that once the causes of cardiogenic pulmonary edema are ruled out (through ultrasound, hemodynamic evaluations—Swan Ganz catheter, electrocardiogram (ECG), and serum markers), we can certify the presence of noncardiogenic pulmonary edema. The cases presented in the specialized literature also mention that noncardiogenic pulmonary edema requires more aggressive invasive ventilation with higher values of PEEP and FiO_2_ [9].

The relevance of the case derives from the fact that based on our previous experience in the clinic in the last 20 years following the appearance of this complication, without the possibility of ECMO, the patient’s evolution was a rapid deterioration and death.

Pulmonary edema after cardiopulmonary bypass during cardiac surgery may be related to a variety of factors, including the effects of cardiopulmonary bypass, anesthesia, hypothermia, surgery, medications, blood transfusions, and inflammatory hyperreactivity [1,2,3]. Considering that the preoperative screening was negative, blood tests showed no signs of infection, and cultures collected both preoperatively and postoperatively were also negative, preoperatory laboratory investigation also pleads for the exclusion of infections (as can be seen in Table 1). The particularity of the case report is the fact that we were able to exclude the infectious component of this pulmonary edema; the patient was not previously diagnosed with any neoplastic disease, noting that the other cases (two in number) that presented this complication had autoimmune disease or neoplasia. The other two cases that developed this complication occurred 13 and 9 years ago, respectively. Both patients died in the operating room, due to the fact that we did not have advanced critical care (ECMO). There are not sufficient data to present them in detail. In these cases, massive pulmonary edema manifested upon separation from cardiopulmonary bypass, with a large amount of yellowish fluid visibly exiting through the intubation tube. Transesophageal echocardiography was performed in both cases, which ruled out left ventricular failure as the underlying cause. One of the cases was diagnosed with renal malignancy. Further investigation is needed to see if there is any correlation between these pathologies. We still cannot know if the cardiac bypass itself triggers an extreme inflammatory reaction or an important autoimmune reaction in specific groups of patients [10]. Last but not least, one of the causes can be neurogenic pulmonary edema and it may appear due to sudden sympathetic activation or other neurological factors [9].

On the 10th day of hospitalization in the ICU, deep vein thrombosis developed which evolved favorably under treatment with heparin. This issue, which is described as frequent following ECMO [11,12,13], is emphasized by clinical research as much more relevant in patients with an aPTT value of less than 60 s [14,15] (Table 5).

Comparing with 11 original articles, it was observed that not all patients who developed pulmonary edema after cardiac surgery had ventricular dysfunction [6,16,17]. According to the cases presented in the literature, noncardiogenic pulmonary edema (NCPE) after cardiac surgery, especially after mitral valve surgery through thoracotomy, occurs both unilaterally (UPE) [18] and bilaterally [16,19,20]. All patients developed NCPE postoperatively in the operating room or immediately after admission to intensive care Also, the cases from the literature that we described benefited from VV-ECMO as the preferred approach [21].

All authors specify that the frequent risk factors in the occurrence of pulmonary edema that can represent a potential trigger are represented by ischemia reperfusion syndrome, prolonged cardiac bypass time without pulmonary ventilation, generalized inflammatory response (Il-6, IL-8, IL-17) [10,22,23,24], documented sepsis (peritonitis, pancreatitis, endocarditis) [9], anesthesia, hypothermia, surgery, medications, blood transfusions, inflammatory hyperreactivity, or maybe even an hyperimmune response [25,26].

The studies also state that all patients benefited from VV-ECMO, being the treatment with the maximum efficiency [27,28,29]. The initiation of ECMO was quick, being imposed by the absence of a response to ARDS-type ventilation and the persistence of liquid externalized from the tracheobronchial tree. The duration of ECMO treatment in these cases varied from 2 to 50 days. Indeed, the cases that required more days of treatment were high-risk patients with a preoperatively EuroScore- and Apache II-calculated mortality of 20–25% and who presented postoperatively other complications besides noncardiogenic pulmonary edema such as diffuse alveolar hemorrhage or bleeding from a different site: pulmonary drainage sites or a tracheostomy site [20]. Other treatments associated with ECMO such as inhaled nitric oxide and high-dose diuretics did not improve oxygenation [17]. All of these results helped to establish the criteria for the initiation of ECMO: partial oxygen pressure/fraction of inspired oxygen < 80 (PaO_2_/FiO_2_ < 80), under FiO_2_ 1.0, PEEP = 18 cmH_2_O, with refractory respiratory acidosis pH < 7.25 [30]. Advanced age is a relative contraindication for ECMO [20]. Differentiation of the two types of cardiogenic and noncardiogenic pulmonary edema is based on clinical judgment, laboratory, imaging investigations, and hemodynamic measurements [9]. The aortic cross-clamping time specified in the literature was between 100 and 265 min. However, these data do not show a correlation with the occurrence of NCPE [2,6,18]. The heterogeneity and diversity of individuals’ responses to the same type of intervention, cardioplegia solution, and aortic cross-clamping period make it difficult to determine a common source of this condition. Of course, the cases evaluated in this research show that longer aortic cross-clamping times have a bigger impact on inflammation, related problems, mortality, and morbidity. Regarding the thoracoscopic approach in cardiac surgery, studies show that the clamping time is longer in thoracoscopic surgery than in median sternotomy, but this surgical approach has multiple advantages, starting with smaller scars, less bleeding, less pain, and a lower risk of infection. For these reasons, in recent years it has been preferred by both surgeons and patients. Many cardiac diseases have been treated in recent years with this type of approach, such as mitral, tricuspid, and aortic valve procedures, congenital heart defects, and cardiac tumors [31]. The type of ventilation during aortic cross-clamping (one ventilated lung or no ventilated lungs) appears to affect the generation of reactive oxidative species [17] but more studies are needed to see the inflammatory impact on the lungs depending on the type of ventilation.

## 5. Conclusions

NCPE after cardiac surgery is a serious complication with high morbidity and mortality. ECMO remains at the moment a promising modality of treatment for the patients who cannot be effectively mechanically ventilated.

The exact cause of the occurrence of NCPE is not yet known, although several factors can predispose to this lung damage. The inflammatory response at the level of the lung parenchyma may be one of the causes, but the triggering factor is not yet fully known. The question remains which patients are more exposed to this complication because even patients without risk factors (no sepsis or massive transfusion, without inflammatory or autoimmune pathologies) developed NCPE after cardiac surgery. Since previous studies have shown that patients with chronic lung disease and pulmonary hypertension exhibited increased levels on pro-inflammatory cytokines, a protocol to dose and investigate the inflammatory profile for patients with cardiac pulmonary hypertension is required.

The inflammatory profile could provide predictability in patients who have an increased risk of developing serious complications. In addition to patients with chronic lung disease, patients with autoimmune diseases and neoplasms who are going to perform cardiac surgery could benefit from the evaluation of the inflammatory profile preoperatively.

## Figures and Tables

**Table 1:** Preoperatory laboratory investigation.

Leukocytes	7.98/10^3^/µL
Erythrocytes	4.24/10^6^/µL
Neutrophils	67.7%
Hemoglobin	13.1 g/dL
Platelets	170/10^3^/µL
Coagulation	aPTT = 30 s, INR = 1.23, PT = 24.2 s
Creatinine	1.45 mg/dL
Alanine aminotransferase (ALT)	44 U/L
Aspartate aminotransferase (AST)	24 U/L
Creatine kinase (CK)	36 U/L
Glucose	102 mg/dL
K^+^	3.8 mmol/L
Na^+^	137 mmol/L
PCT	<0.5 µg/L
High-sensitivity C-reactive protein	4 mg/L

**Table 2:** 22.03.2024 (Day 1 of VV-ECMO).

h	18:30	20:00	21:00	22:00	05:30
Flow (L/min)	2.8 → 3.05	3.05 → 3.31	3.31 → 3.15	3.05	3.05
RPM	2000 → 2200	2200 → 2350	2350 → 2255	2255	2300
P_ven_ (mmHg)	−95	−98	−95	−95	−94
P_art_ (mmHg)	10	28	18	17	19
P_int_ (mmHg)	26	45	34	35	43
ΔP	16	17	18	18	19
S_v_O_2_ (%)	68.5	57.3	66.5	72	72.9
FiO_2_ ECMO (%)	100	100	100	100	100
O_2_ flow (L/min)	4	4	4 → 3	3	3
pH	7.19	7.18	7.26	7.27	7.32
Pa_CO2_ (mmHg)	49	45	36.7	44.1	39.5
Pa_O2_ (mmHg)	97.8	57.4	85.5	100.2	99.4
Hb (g/dL)	10.1	10.4	11	11.2	12.5
Lactate (mmol/L)	11.2	11.5	12	11.4	7.8
AP (mmHg)	90/60	130/64	122/63	125/67	120/66
VM	P-SIMV → APRV	APRV	APRV	APRV	APRV
**FiO_2_ (%)**	50	50	50	50	50
**VT (mL)**	200 → 100	275	150	330	300
**RR**	20 → 10	10	10	10	10
**PEEP (cm_H2O_)**	10	10	10	10	10
**P_plat_**	24	24	24	22	22
**P_peak_**	24	24	24	22	22
**Compliance (mL/cm_H2O_)**	8	21	10	24	26
**Resistance (cm_H2O_/L/s)**	76	26	36	12	10

**Table 3:** Postoperative analyses, patient on ECMO and treatment with continuous-dose heparin under aPTT control.

	ECMO Day 1	ECMO Day 2	ECMO Day 3	ECMO Day 4	ECMO-Decannulation Day 5
Leukocytes/10^3^/µL	23.89	25.77	23.34	17.86	12.42
Erythrocytes/10^6^/µL	4.14	4.41	3.26	3.32	3.5
Neutrophils%	93.4%	93.1	92.6	90.5	88.1
Hemoglobin g/dL	12.7	10.5	10	10.2	10.4
Platelets/10^3^/µL	152/10	109	79	55	72
Coagulation					
aPTT s	74	55	50	37.1	31.8
INR	1.77	1.91	1.6	1.49	1.35
PT s	27.8	28.5	28	23.7	22.1
D-Dimer ng/mL	700	1280	1500		
Creatinine mg/dL	1.94	2.12	2.03	1.75	1.41
Urea mg/dL	63	82	96	85	100
ALT mg/dL	37	36	30	33	32
AST U/L	81	69	52	41	33
CK U/L	1158	1227	825	678	495
CK-MB U/L	80	43	23	17	13
LDH U/L	424	437	446	473	465
High-sensitivity C-reactive protein mg/L	58.9	229.5	310	262	274

**Table 4:** The inflammatory response after surgery.

	Preoperative	ECMO Day 1	ECMO Day 2	ECMO Day 3	ECMO Day 4	ECMO Day 5—Decannulation Day
**High-sensitivity C-reactive protein mg/L**	<5	58.9	229.5	310	262	274
**Procalcitonin**	negative	1.7	2.3	5	3.7	2.4
**Neutrophil** **lymphocyte ratio**	4.06	71	40	23		
**IL-6**		429.35	401	337	194	86
**IL-17**	0		0.0044			

**Table 5:** The risk of deep vein thrombosis correlated with aPTT.

	aPPT > 60s	aPPT < 60 s
Bleeding episodes	56%	8%
Clotting events	7%	32%

## Supplementary Materials

The following supporting information can be downloaded at https://www.mdpi.com/article/10.3390/diseases12120316/s1, Table S1: Parameters of ECMO and ventilation on weaning day.

## References

[1] Wang S., Liang P. (2023). Acute Pulmonary Edema Following Cardiopulmonary Bypass: A Case Report. Asploro J. Biomed. Clin. Case Rep..

[2] Arima T., Tatebayashi T., Noji S. (2023). Management of fulminating non-cardiogenic pulmonary edema following cardiac surgery. J. Surg. Case Rep..

[3] Zeng J., Li Y., Liu J., Li L. (2023). Severe noncardiogenic pulmonary edema after cardiopulmonary bypass. Helyon.

[4] Cui Y., Zhang Y., Dou J., Shi J., Zhao Z., Zhang Z., Chen Y., Cheng C., Zhu D., Quan X. (2022). Venovenous vs. Venoarterial Extracorporeal Membrane Oxygenation in Infection-Associated Severe Pediatric Acute Respiratory Distress Syndrome: A Prospective Multicenter Cohort Study. Front Pediatr..

[5] Xu W.-J., Wu Q., He W.-N., Wang S., Zhao Y.-L., Huang J.-X., Yan X.-S., Jiang R. (2023). Interleukin-6 and pulmonary hypertension: From physiopathology to therapy. Front. Immunol..

[6] Goyal S., Dashey S., Zlocha V., HannaJumma S. (2019). The successful use of extra-corporeal membrane oxygenation as rescue therapy for unilateral pulmonary edema following minimally invasive mitral valve surgery. Perfusion.

[7] Rong L.Q., Di Franco A., Gaudino M. (2016). Acute respiratory distress syndrome after cardiac surgery. J. Thorac. Dis..

[8] Coppola S., Caccioppola A., Froio S., Ferrari E., Gotti M., Formenti P., Chiumello D. (2019). Dynamic hyperinflation and intrinsic positive end-expiratory pressure in ARDS patients. Crit Care.

[9] Meyer T.E., Shih J.A., Harrington C. (2019). Acute Heart Failure and Pulmonary Edema. Cardiac Intensive Care.

[10] Tsakiridis K., Mpakas A., Kesisis G., Arikas S., Argyriou M., Siminelakis S., Zarogoulidis P., Katsikogiannis N., Kougioumtzi I., Tsiouda T. (2014). Lung inflammatory response syndrome after cardiac-operations and treatment of lornoxicam. J Thorac. Dis..

[11] Abruzzo A., Gorantla V., Thomas S.E. (2022). Venous thromboembolic events in the setting of extracorporeal membrane oxygenation support in adults: A systematic review. Thromb. Res..

[12] Gu Y., Bjelic M., Panda K., Usman A.A., Magnuson R., Gosev I. (2024). Cannula-Associated Deep Vein Thrombosis After Venovenous Extracorporeal Membrane Oxygenation in Patients with and Without Systemic Anticoagulation. J. Cardiothorac. Vasc. Anesth..

[13] Sklar M.C., Sy E., Lequier L., Fan E., Kanji H.D. (2016). Anticoagulation Practices during Venovenous Extracorporeal Membrane Oxygenation for Respiratory Failure. A Systematic Review. Ann. Am. Thorac. Soc..

[14] Kakkos S.K., Gohel M., Baekgaard N., Bauersachs R., Bellmunt-Montoya S., Black S.A., ten Cate-Hoek A.J., Elalamy I., Enzmann F.K., Geroulakos G. (2021). Editor’s Choice—European Society for Vascular Surgery (ESVS) 2021 Clinical Practice Guidelines on the Management of Venous Thrombosis. Eur. J. Vasc. Endovasc. Surg..

[15] Vahanian A., Beyersdorf F., Praz F., Milojevic M., Baldus S., Bauersachs J., Capodanno D., Conradi L., De Bonis M., De Paulis R. (2022). 2021 ESC/EACTS Guidelines for the management of valvular heart disease. EuroIntervention.

[16] Culliford A.T., Thomas S., Spencer F.C. (1980). Fulminating noncardiogenic pulmonary edema. A newly recognized hazard during cardiac operations. J Thorac Cardiovasc Surg..

[17] Vardas P.N., Matthews C., Rosati C.M., Beckman D.J. (2019). Severe re-expansion pulmonary edema after conventional cardiac surgery: Identification and management. J. Card. Surg..

[18] Kitahara H., Okamoto K., Kudo M., Yoshitake A., Hayashi K., Inaba Y., Ai K., Suzuki T., Morisaki H., Shimizu H. (2015). Successful management of severe unilateral re-expansion pulmonary edema after mitral valve repair with mini-thoracotomy using extracorporeal membrane oxygenation. Gen. Thorac. Cardiovasc. Surg..

[19] Bignami E., Frati E., Meroni R., Verzini A., Pozzoli A., Benussi S., Alfieri O. (2014). Extracorporeal venovenous membrane oxygenation in the treatment of respiratory insufficiency following cardiac surgery. J. Card. Surg..

[20] Takagaki M., Yamaguchi H., Ikeda N., Takeda K., Kasai F., Yahagi K., Kanzaki S., Mitsuyama S., Kadowaki T., Kotani T. (2018). Post-cardiotomy venovenous extracorporeal membrane oxygenation without heparinization. Gen. Thorac. Cardiovasc. Surg..

[21] Cavanaugh N.B., Nguyen L.H., Arora L., Singhal A.K., Hanada S. (2024). Emergent veno-venous extracorporeal membrane oxygenation during aortic valve replacement following severe re-expansion pulmonary edema: A case report. SAGE Open Med. Case Rep..

[22] Keskinidou C., Vassiliou A., Dimopoulou I., Kotanidou A., Orfanos S. (2022). Mechanistic Understanding of Lung Inflammation: Recent Advances and Emerging Techniques. J. Inflamm. Res..

[23] Pourfathi M., Cereda M., Chatterjee S., Xin Y., Kadlecek S., Duncan I., Hamedani H., Siddiqui S., Profka H., Ehrich J. (2018). Lung Metabolism and Inflammation during Mechanical Ventilation; An Imaging Approach. Sci Rep..

[24] Faustino L.D. (2022). Chapter 10—Lungs—Inflammatory and respiratory system. Macrophages in the Human Body.

[25] Gopallawa I., Dehinwal R., Bhatia V., Gujar V., Chirmule N. (2023). A four-part guide to lung immunology: Invasion, inflammation, immunity, and intervention. Front Immunol..

[26] Burgoyne R.A., Fisher A.J., Borthwick L.A. (2021). The Role of Epithelial Damage in the Pulmonary Immune Response. Cells.

[27] Grotberg J.C., Reynolds D., Kraft B.D. (2023). Management of severe acute respiratory distress syndrome: A primer. Crit. Care.

[28] Muenster S., Nadal J., Schewe J.-C., Ehrentraut H., Kreyer S., Putensen C., Ehrentraut S.F. (2024). Analysis of Patients with Severe ARDS on VV ECMO Treated with Inhaled NO: A Retrospective Observational Study. J. Clin. Med..

[29] Bullen E.C., Teijeiro-Paradis R., Fan E. (2020). How I Select Which Patients with ARDS Should Be Treated with Venovenous Extracorporeal Membrane Oxygenation. Chest.

[30] Nakamura H., Yamaguchi H., Amano A., Nakao T. (2013). Venovenous extracorporeal membrane oxygenation is effective against post-cardiotomy acute respiratory failure in adults. Gen. Thorac. Cardiovasc. Surg..

[31] Jiang Q., Huang K., Zhao D., Xiao Y., Ma X., Hu S. (2024). Innovations and Developments in Totally Thoracoscopic Cardiac Procedures. Heart Surg. Forum.

