# Supplementary material for: ECMO in the Management of Noncardiogenic Pulmonary Edema with Increased Inflammatory Reaction After Cardiac Surgery: A Case Report and Literature Review
Source: Diseases. 2024 Dec 4;12(12):316. doi: 10.3390/diseases12120316 (PMC11727641; doi:10.3390/diseases12120316)
Supplement: Supplementary file 1 [file diseases-12-00316-s001.zip › diseases-3271993-supplementary.pdf]

Supplementary material for the article “”

Table S1. Parameters of ECMO and ventilation on weaning day.

	06:00	10:00	10:45	11:30	13:00	14:00	15:00	16:00	17:00	18:00
Debit (L/min)	3.2	3.25	3.2	3.2 -> 2	2	1.9	1.9	1.9	1.9	1.9
RPM	2330	2330	2330	2330 -> 1670	1670	1630	1630	1630	1630	1630
Pven (mmHg)	-90	-92	-91	-70	-70	-65	-66	-70	-64	-64
Part (mmHg)	26	23	21	-13	-12	-16	-14	-15	-12	-11
Pint (mmHg)	40	40	39	-5	-2	-1	-2	-3	1	-1
Delta p (mmHg)	19	20	19	12	12	11	12	12	10	11
SvO2 (%)	66.2	64,5	73.2	60	64.5	67.6	64.5	58.3	63	63.8
FiO2 ECMO (%)	100	100	100	100	100	100	100	100	100	100
Flux O2 (L/min)	2.5	2.5	2.5	2.5 -> 2	2 -> 1.5	1.5 -> 1	1 -> 0.5	0.5 -> 0	0	0
pH	7.43	7.49	7.48	7.49	7.49	7.48	7.43	7.43	7.42	7.42
pCO2 ()mmHg	39.8	32.9	33.6	32.3	32.1	33.4	39.4	39.6	41.3	39.9
pO2 (mmHg)	121.5	88.9	433.7	161.4	155.5	159.9	145.1	109.7	83.2	109.1
Hb (g/dL)	9.8	9.9	9.6	9.6	9.2	9.2	10	9.8	9.7	9.5
Lactate (mmol/L)	1.6	1.3	1.3	1.3	1.4	1.5	1.5	1.4	1.3	1.3
TA (mmHg)	133/64	125/64	136/69	138/66	120/60	118/58	111/55	105/52	135/64	142/68
VM	APRV	APRV -> P-SIMV	P-SIMV	P-SIMV	P-SIMV	P-SIMV	P-SIMV	P-SIMV	P-SIMV	P-SIMV
FiO2 (%)	30	30 -> 100	100 -> 50	50	50	50	50	50	50	50
VT (ml)	414	440	550	430	425	400	530	520	570	570
RR	10	10 -> 12	12	12	12	12	12	12	12	12
PEEP (cmH2O)	10	10 -> 9	9	9	9	9 -> 8	8->7	7	7	7
Pplat (cmH2O)	20	20 -> 23	23	21	21	20	17	18	17	17
Ppeak (cmH2O)	20	20 -> 23	23	21	21	20	18	18	18	18
Compliance (ml / cmH2O)	40	32	38	34	36	35	56	53	54	54
Resistentace (cmH2O/L/s)	9	9	10	10	9	9	10	6	8	8
